# Quantification of turbulence and velocity in stenotic flow using spiral three‐dimensional phase‐contrast MRI

**DOI:** 10.1002/mrm.25698

**Published:** 2015-04-04

**Authors:** Sven Petersson, Petter Dyverfeldt, Andreas Sigfridsson, Jonas Lantz, Carl‐Johan Carlhäll, Tino Ebbers

**Affiliations:** ^1^ Division of Cardiovascular Medicine Department of Medical and Health Sciences Linköping University Linköping Sweden; ^2^ Center for Medical Image Science and Visualization (CMIV), Linköping University Linköping Sweden; ^3^ Division of Media and Information Technology Department of Science and Technology/Swedish e‐Science Research Centre (SeRC) Linköping University Linköping Sweden; ^4^ Department of Clinical Physiology Karolinska Institutet and Karolinska University Hospital Stockholm Sweden; ^5^ Department of Clinical Physiology Institution of Medicine and Health Sciences, Linköping University Linköping Sweden

**Keywords:** phase contrast mri, 4d flow, turbulence mapping, spiral, stenosis

## Abstract

**Purpose:**

Evaluate spiral three‐dimensional (3D) phase contrast MRI for the assessment of turbulence and velocity in stenotic flow.

**Methods:**

A‐stack‐of‐spirals 3D phase contrast MRI sequence was evaluated in vitro against a conventional Cartesian sequence. Measurements were made in a flow phantom with a 75% stenosis. Both spiral and Cartesian imaging were performed using different scan orientations and flow rates. Volume flow rate, maximum velocity and turbulent kinetic energy (TKE) were computed for both methods. Moreover, the estimated TKE was compared with computational fluid dynamics (CFD) data.

**Results:**

There was good agreement between the turbulent kinetic energy from the spiral, Cartesian and CFD data. Flow rate and maximum velocity from the spiral data agreed well with Cartesian data. As expected, the short echo time of the spiral sequence resulted in less prominent displacement artifacts compared with the Cartesian sequence. However, both spiral and Cartesian flow rate estimates were sensitive to displacement when the flow was oblique to the encoding directions.

**Conclusion:**

Spiral 3D phase contrast MRI appears favorable for the assessment of stenotic flow. The spiral sequence was more than three times faster and less sensitive to displacement artifacts when compared with a conventional Cartesian sequence. Magn Reson Med 75:1249–1255, 2016. © 2015 Wiley Periodicals, Inc.

## INTRODUCTION

Stenotic flow is often characterized by high‐velocity jets, high acceleration, and disturbed or turbulent flow fluctuations. These turbulent flow fluctuations drastically decrease the transport efficiency of the blood due to viscous dissipation, which is the major cause of pressure drop over a constriction. Exposure of biological tissue to abnormal turbulent stresses can also cause tissue damage, such as mechanical damage of blood constituents resulting in hemolysis and compromised hemostasis 1 and endothelial dysfunction 2.

Time‐resolved three‐dimensional (3D) phase‐contrast (PC) MRI, referred to as 4D flow MRI, is a powerful tool for the quantification of a range of hemodynamic parameters in stenotic flow, such as flow eccentricity, turbulent kinetic energy (TKE) and pressure drop 3, 4, 5, 6, 7. However, the application of 4D flow MRI is limited by long scan times and many PC‐MRI artifacts are more prominent in stenotic flow. High velocities and acceleration may result in spatial misregistration (displacement) artifacts. Furthermore, disturbed and turbulent flow can cause flow‐related signal loss due to intravoxel phase dispersion 8, and ghosting due to view‐to‐view variations. This signal loss can lead to inaccurate flow estimates, but can be decreased by usage of shorter echo times (TE) 8. Ultrashort TE PC‐MRI have been shown to reduce artifacts such as signal loss, as well as to increase the accuracy of flow quantification of stenotic flow 9, 10, 11, but it does not decrease the long scan times.

Spiral readouts starts in the center of k‐space, leading to a shorter TE and less T2* signal decay in the center of k‐space, which is advantageous in the assessment of stenotic flow. These trajectories are also very efficient, as a large part of the repetition time (TR) can be spent on actually reading data. In aortic 4D flow MRI, spiral readouts have been shown to reduce the scan time of aortic 4D flow MRI by a factor of two to three compared with a conventional Cartesian measurement (with a SENSE factor of two), without reducing the data quality for pathline analysis and measurement of cardiac output 12. Spiral trajectories have previously been shown to be suitable for 2D PC‐MRI of high‐speed flow jets 13 and unsteady flow systems 14. A recent study, evaluated spiral 4D PC‐MRI and found that the short TE of spiral trajectories are advantageous when assessing maximum velocity and flow rate in stenotic nonpulsatile flow in vitro 15. However, the effect of spiral sampling on the assessment of TKE has not yet been investigated.

The aim of this work was to evaluate spiral three‐directional 3D PC‐MRI for the assessment of turbulence and velocity in stenotic flow. A 3D PC‐MRI stack‐of‐spirals sequence was evaluated in an in vitro flow phantom against a conventional Cartesian sequence and computational fluid dynamics (CFD) data. The assessment of TKE, volume flow rate, maximum velocity, and the effect of displacement artifacts were compared between Cartesian and spiral acquisition techniques.

## METHODS

Both spiral and Cartesian imaging were performed using three different scan orientations and four different flow rates in an in vitro flow model of stenotic flow, as described below. Numerical flow data resolving the turbulent velocities were obtained using CFD simulations.

### In Vitro Flow Phantom

Measurements of steady flow were made in an in vitro flow phantom consisting of a straight rigid plastic pipe with an inner diameter of 14.6 mm, and a 75% area reduction stenosis 16, 17. The entrance length was approximately 100 diameters. A gear pump (Gearchem G6, Pulsafeeder, Rochester, NY) was connected to the phantom by means of plastic hoses. The pump was fed by a computer‐controlled AC servo motor (JVL Industri Elektronik A/S, Blokken, Denmark). A honeycomb flow straightener was placed at the inlet to reduce flow structures. The fluid used was water at 23°C. Water‐filled bottles were placed around the phantom to improve coil loading.

Measurements were performed at volume flow rate 10 mL/s, 20 mL/s, 56 mL/s, and 112 mL/s, resulting in a jet velocity of approximately 0.3 m/s, 0.6 m/s, 1.4 m/s, and 2.8 m/s, respectively.

### Acquisition and Postprocessing

A clinical 1.5 Tesla (T) MRI system (Achieva, Philips, Best, The Netherlands) with 33 mT/m gradient strength and 180 T/m/s slew rate was used for all measurements. The spiral sequence consisted of a stack of spirals, and every slice consisted of 12–15 spiral interleaves. Conventional Cartesian scans with identical spatial resolution were used for comparison.

The effect of scan orientation was assessed by using three different orientations: (i) coronal (COR) with frequency and spiral encoding in the flow direction, (ii) transverse (TRA) with slice encoding in the flow direction, and (iii) oblique (OBL) orientation with frequency and slice encoding in the flow direction (45°). As the viscosity of water is low compared with blood, physiological turbulent velocity fluctuations and physiological velocities could not be achieved simultaneously. Therefore, at flow rates 10 and 20 mL/s, which correspond to Reynolds numbers 1000 and 2000, respectively, two velocity encoding ranges (VENC) were used: one was optimized for turbulence mapping, and a second higher VENC was optimized for velocity mapping without aliasing. The low VENCs resulted in longer TE and repetition time (TR) as the maximum gradient strength was already reached. At flow rates 56 and 112 mL/s, which resulted in maximum velocities corresponding to clinically slightly increased aortic velocity (increased‐flow) and mild to moderate aortic stenosis (stenotic‐flow), only velocity mapping and assessment of displacement artifacts were carried out. Scan parameters for all scans are shown in Table 1. Gradient spoiling was used for all sequences in the phase‐encoding directions and no RF‐spoiling was used. The TE in the spiral velocity measurements for the two higher VENCs was 2.1–2.4 ms shorter than the TE for the Cartesian measurements.

**Table 1:** MRI Scan Parameters.

Scan	VENC [cm/s]	Scan time [min]	No. of readouts	TE [ms]	TR [ms]	Interleaves × spiral duration [ms]	Matrix	Voxel size [mm]	Flip angle
Spiral COR	10	1:36	285	5.9	14.1	15×5.5	112×112×15	1.5	9.7
	35	1:21		3.7	11.9				
	70	1:16		3.0	11.2				
	150	0:49		2.4	10.5				
	300	0:48		2.1	10.3				
Spiral TRA	10	11:40	1224	5.9	14.5	12×6	96×96×80	1.5	9.7
	35	8:44		3.7	12.3				
	70	5:34		2.9	11.5				
	150	2:40		2.3	10.9				
	300	2:34		1.9	10.5				
Spiral OBL	150	2:40	1224	2.3	10.9	15×5.5	96×96×80	1.5	9.7
	300	2:34		1.9	10.5				
Cart. COR	10	7:31	1634	8.1	11.4	‐	112×112×15	1.5	8.5
	35	5:51		5.5	8.9				
	70	5:29		4.9	8.2				
	150	3:31		4.5	7.9				
	300	3:29		4.5	7.8				
Cart. TRA	10	22:31	4284	8.2	11.6	‐	80×80×80	1.5	8.5
	35	18.14		5.7	9.1				
	70	16:43		4.9	8.3				
	150	6:48		4.5	7.9				
	300	6:41		4.3	7.7				
Cart. OBL	150	6:48	4284	4.5	7.9	‐	80×80×80	1.5	8.5
	300	6:41		4.3	7.7				

For each velocity scan, an identical scan was carried out with the pump turned off. The velocities from these scans were smoothed using a spatial filter and then subtracted from the velocity data so as to correct for velocity offsets. A total three to seven signal averages were used to boost SNR.

### Numerical Flow Data

Numerical flow data was used to evaluate eventual discrepancies between spiral and Cartesian sampling. The flow inside the phantom for Reynolds numbers 1000 and 2000 was simulated numerically by solving the Navier‐Stokes equations in ANSYS CFX 14.0. A computational mesh was made in ANSYS ICEM 14.0 and contained 10 million high quality anisotropic hexahedral cells. The nondimensional wall distance Y+ was always less than one to ensure a good near‐wall resolution, and the thickness of the mesh cells close to the wall grew exponentially by a factor of 1.05 until it matched the mesh size in the center of the phantom. Turbulent flow fluctuations were resolved using Large Eddy Simulation (LES), a technique that resolves the larger energy‐carrying turbulent scales and models the smaller isotropic scales where energy dissipation occurs. The simulation used the WALE sub‐grid scale model 18, and the numerical schemes were second order accurate. The time step was 5·10^−5^ s, which has been shown to be sufficient for these kinds of flow 18, 19, 20, and also gave a Courant number of less than one. The convergence criterion was 1·10^−6^ and global imbalances of mass and momentum were always less than 0.1. Sampling of flow statistics was started after initial transient effects had disappeared. Results were extracted along the centerline after the results had converged.

Velocity profiles from the MRI measurements were prescribed at the inlet of the model as boundary conditions, while the outlet used a constant static pressure. The walls were considered rigid and to obey the no‐slip condition. The fluid was water with a constant density of 997 [kg/m^3^] and dynamic viscosity of 8.899·10^−4^ [kg m^−1^ s^−1^].

### Data Analysis

Turbulent kinetic energy can be computed from intravoxel velocity standard variation (IVSD), which can be obtained from the flow‐induced signal loss 16, 21, 22. Estimates of IVSD from 4D flow MRI with Cartesian sampling have been shown to agree well with laser Doppler anemometry measurements, particle image velocimetry, and computer fluid dynamics (CFD) simulations of both in vitro and in vivo flow 17, 20, 23, 24. The TKE per voxel was computed from the IVSD in the three directions as

(1) $$TKE=\frac{\rho }{2}(IVSD_{X}^{2}+IVSD_{Y}^{2}+IVSD_{Z}^{2})$$

where ρ is the density of water. The total TKE in the post‐stenotic region of the phantom was obtained by integrating the TKE in the phantom between the center of the stenosis (X = 0) and six diameters downstream (X = 6).

The velocities and turbulent kinetic energy were interpolated along the centerline of the phantom. Maximum velocities were obtained by finding the maximum speed in a segmentation of the phantom. To make the maximum velocity estimates less noise dependent the speed data was first smoothed using a Gaussian kernel, which was five voxels wide (the inner diameter of the phantom is 9.7 voxels wide). The TKE estimates were also evaluated by computing the root‐mean‐square error (RMSE) with the Cartesian TRA measurement as reference. As the FOV is slightly different between the two methods and the two orientations, it was necessary to interpolate all data to the Cartesian TRA grid to perform voxel‐to‐voxel comparison.

The volume flow rates were computed from a segmentation of the through‐plane velocity at cross‐sectional planes of the phantom. An automatic segmentation was based on the true radius of the vessel. This segmentation was then controlled and, if necessary, edited manually to fit the magnitude data. According to the continuity equation, the flow rate should be equal along the phantom.

## RESULTS

The estimated maximum velocities were in the interval of 1.37 to 1.46 m/s and 1.34 to 1.39 m/s for the spiral and Cartesian measurements of the increased‐flow case, respectively (Table 2). For the stenotic‐flow case, the maximum velocities were 2.69 to 2.75 m/s and 2.66 to 2.87 m/s for the spiral and Cartesian measurements, respectively (Table 2).

**Table 2:** Maximum Velocity and Total Turbulent Kinetic Energy^a^

Max velocity [m/s]	Increased‐flow	Stenotic‐flow
Spiral TRA	1.37	2.70
Cartesian TRA	1.39	2.67
Spiral COR	1.39	2.69
Cartesian COR	1.35	2.66
Spiral OBL	1.46	2.75
Cartesian OBL	1.34	2.87
Total TKE [J]	Re. 1000	Re. 2000
Spiral TRA	2.08*10^−5^	9.56*10^−5^
Cartesian TRA	2.03*10^−5^	9.36*10^−5^
Spiral COR	1.96*10^−5^	8.71*10^−5^
Cartesian COR	1.94*10^−5^	7.79*10^−5^
CFD	1.88*10^−5^	6.82*10^−5^

a The VENC was 150 and 300 cm/s for the increased‐flow and stenotic‐flow cases, respectively. The VENC was 10 and 35 cm/s for Reynolds number 1000 and 2000, respectively. The TKE was integrated between the center of the stenosis and six diameters downstream.

**Figure 1:**
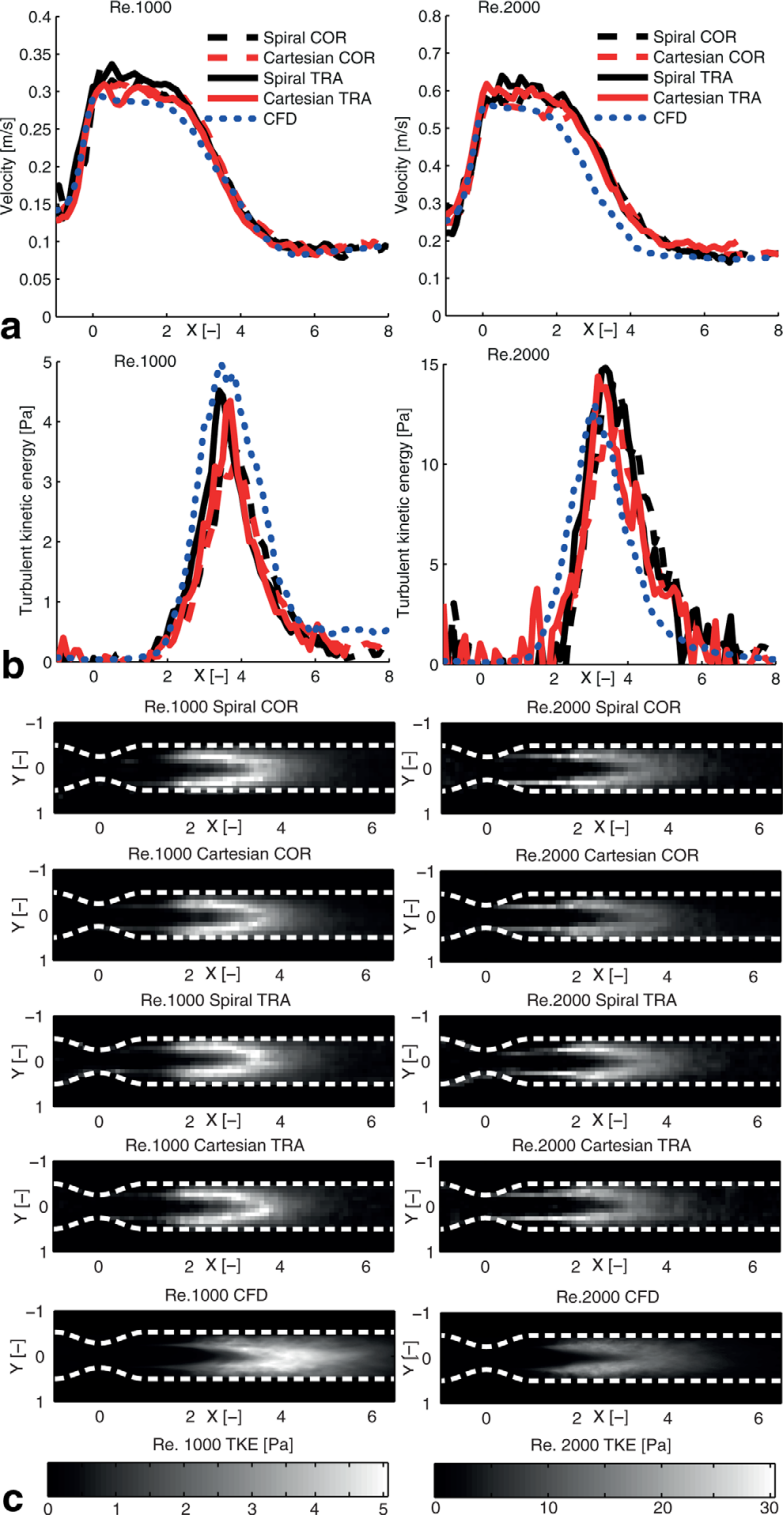
The velocity (**a**) and turbulent kinetic energy (**b**) along the centerline of the phantom together with cross‐sectional images (**c**) of the turbulent kinetic energy for Reynolds number 1000 (left) and 2000 (right) from Spiral and Cartesian 3D PC‐MRI, for two different orientations, coronal (COR) and transverse (TRA), as well as CFD data. The VENC was 35 and 10 cm/s for the velocity and turbulence mapping, respectively. X and Y denote distance from the center of the stenosis normalized by the unconstructed pipe diameter (14.6 mm). The principal flow direction is in the positive X‐direction.

The spiral and Cartesian measurements resulted in similar total TKE for both Reynolds numbers, even though the COR orientation resulted in slightly lower estimates compared with TRA for both methods (Table 2). The total TKE from the CFD data was slightly lower than the MRI based estimates. Moreover, the TKE along the centerline of the phantom matched well between the spiral and Cartesian measurements for all combinations of Reynolds numbers and orientations (Fig. 1b). Overall, the spiral and Cartesian TKE data agreed well with the CFD TKE data. Visual inspection of TKE images also showed good agreement and no apparent discrepancies between the spiral and Cartesian data (Fig. 1c). For Reynolds number 1000, the RMSE from the comparison with Cartesian TRA was 0.52 and 0.39 Pa for the spiral and Cartesian COR measurements, respectively. For Reynolds number 2000, the RMSE from the comparison with Cartesian TRA was 3.21 and 3.26 Pa for the spiral and Cartesian COR measurements, respectively. For spiral TRA, the RMSE was 0.41 and 3.22 for Reynolds number 1000 and 2000, respectively.

**Figure 2:**
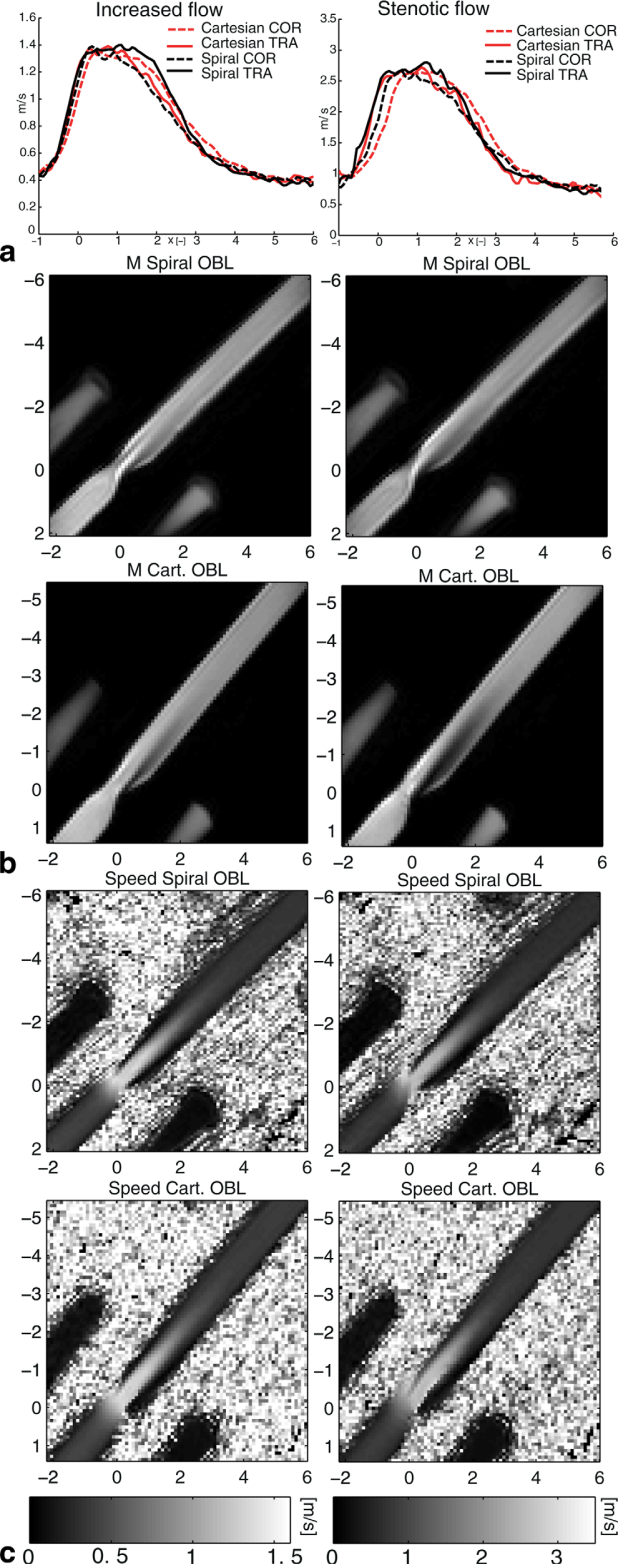
**a**: Plots of the velocity along the centerline of the phantom of the increased‐flow case (left) and stenotic‐flow case (right), respectively. Both Cartesian and spiral velocity data from the coronal (COR) and transverse (TRA) orientations are shown. The principal flow direction is in the positive X‐direction. Images of magnitude (**b**) and speed (**c**) from the oblique (OBL) orientation of the increased‐flow case (left) and stenotic‐flow case (right). The VENC was 150 and 300 cm/s for the increased‐flow case and stenotic‐flow case, respectively. X denotes the distance from the center of the stenosis normalized by the unconstructed pipe diameter (14.6 mm). For the OBL orientation, frequency and slice encoding was carried out along the vertical and horizontal axis, respectively.

Displacement was smaller for the spiral COR measurement compared the Cartesian COR, which was especially apparent at the site of flow acceleration (Fig. 2a). In the spiral and Cartesian OBL measurements, displacement can be seen in magnitude and flow images for both methods, but is less prominent in the spiral measurement (Fig. 2b,c). The stenosis is displaced toward the upper left corner of the images.

**Figure 3:**
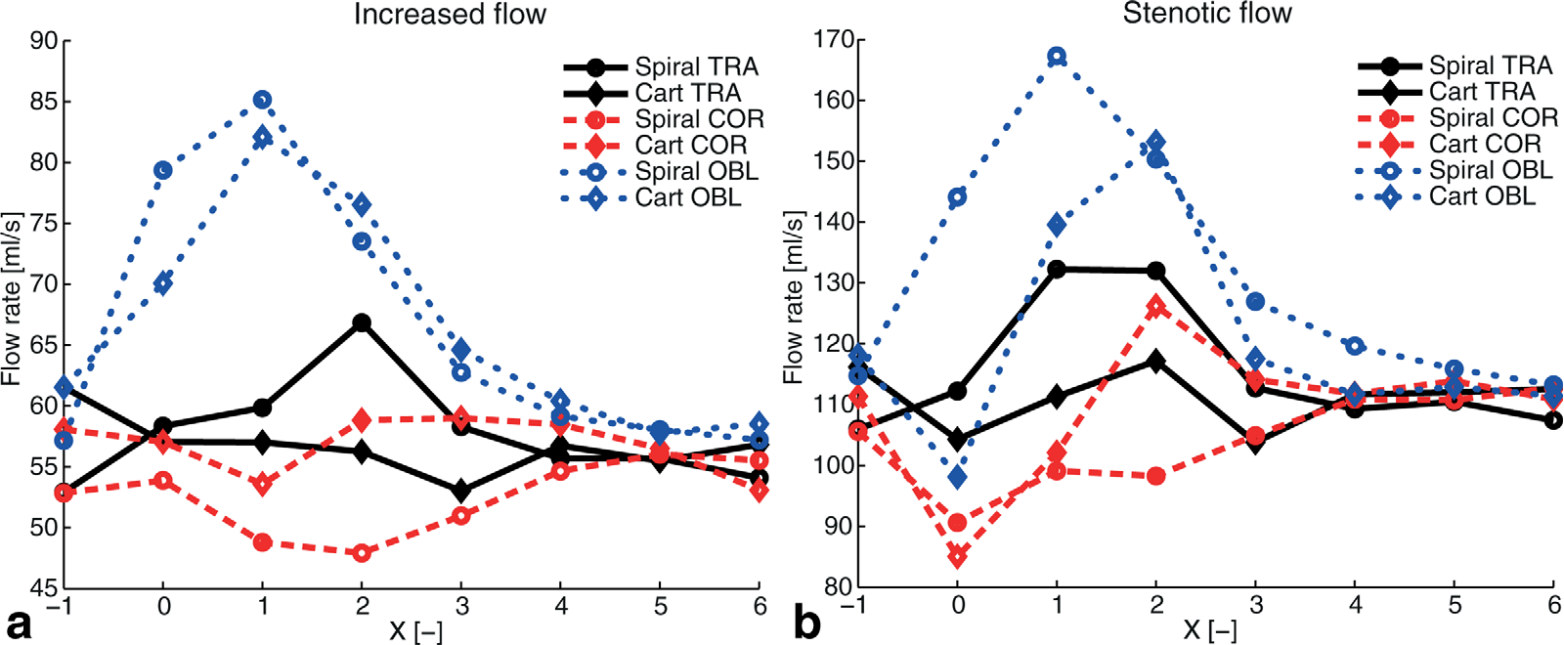
The volume flow rate from the increased‐flow case (**a**) and the stenotic‐flow case (**b**) for the different orientations. The VENC was 150 and 300 cm/s for these two flow cases, respectively. X denotes position of the cross sectional plane from which the flow rate was computed and is the distance from the center of the stenosis normalized by the unconstructed pipe diameter (14.6 mm). The principal flow direction is in the positive X‐direction. Nominal flow rate should be 56 mL/s in the increased‐flow case and 112 mL/s in the stenotic‐flow case, for all planes.

Flow rate estimates were rather consistent upstream of the stenosis as well as after 4 diameters downstream of the stenosis. However, over the jet, the flow rate estimates varied more, especially for the OBL orientations where around 50% higher values were obtained (Fig. 3).

## DISCUSSION

Spiral 3D PC‐MRI was evaluated for the assessment of stenotic flow in vitro. The TKE velocity, and volume flow rate from the spiral and Cartesian measurements agreed well for all flows. Displacement artifacts were less prominent in the spiral measurements and the scan time of the spiral sequence was 1/3 of the Cartesian sequence.

Maximum velocity estimates from the different orientations and sequences show relatively small differences. In 2D through‐plane velocity measurements, the maximum velocity is underestimated if the flow jet is not perpendicular to the plane 13. This problem is not expected in our study, as we used a three‐dimensional and three‐directional velocity measurement. Instead, we see a slight increase of the measurement of the maximum velocity in the jet for the oblique orientation. Measurements with spiral readouts on a similar phantom have shown that a TE longer than 3 ms will result in less accurate maximum velocity estimates 15. In the present work, TE was 1.9–2.5 ms and 4.3–4.5 ms for the spiral and Cartesian maximum velocity measurements, respectively.

The flow rate estimates from the spiral and Cartesian measurements agreed well. However, the flow rate estimates seem to be less accurate when the estimation is performed in the region of the post‐stenotic flow jet, especially for the OBL orientations. This corresponds well to previous observations of flow rate measured with 4D flow spiral measurements in jet regions 15. For 2D through‐plane PC‐MRI, a TE longer than 3 ms has been shown to cause severe underestimation of flow rate for actual flow rates over 300 mL/s 8. Moreover, the peak flow rate in pulsatile stenotic flow was shown to be underestimated using a 4D flow sequence with a TE of 3.9 ms for peak flow rates over 250 mL/s, while for an ultrashort TE sequence with a TE of around 1 ms no underestimation was seen 11. No apparent underestimation was observed in this work for flows up to 112 mL/s. Flow rate estimates from the spiral measurements may be influenced by misregistration of low and high frequencies for moving objects, which can occur when the low frequencies (center of k‐space) are acquired before the high 25, 26. However, this misregistration would be most prominent for regions represented by high frequencies in k‐space, and those regions mainly exist at physical boundaries of the flow, which are characterized by low velocities 14. Also, the short spiral readouts used in this work reduce this misregistration effect. The spiral readouts also result in a time‐varying first gradient moment, which causes the velocity encoding to vary across k‐space. This effect increases toward the periphery of k‐space and results in a broadened point‐spread function for moving objects 26, 27. The flow jet is surrounded by back flow, potentially resulting in partial volume artifacts in the boundary between the jet and the backflow, and this may have contributed to the decreased accuracy in the flow‐rate estimates.

There was good overall agreement between the TKE from CFD and the two different MRI methods. However, the simulated CFD data deviated slightly from the measured flow, likely due to the sensitivity of inlet conditions in the simulation of turbulent flow. Small changes of the inlet conditions may have a large influence on the resulting data. No apparent differences between the spiral and Cartesian TKE estimates were found. The total TKE from the spiral and Cartesian measurements agreed well, although there were some discrepancies between the COR and TRA orientations. The total TKE in the post‐stenotic region from the CFD data was lower than the total TKE estimated from the MRI measurements. Additional contributions from noise and shear stresses in the IVSD estimation as well as deviations between simulated and measured flow may have contributed to the difference in total TKE. The TKE contribution in the MRI data from the shear stresses surrounding the jet is seen most clearly for Reynolds number 2000 (Fig. 1c), while no elevated TKE values in this region are seen in the CFD data. The RMSE values with Cartesian TRA as reference were around 10% of the maximum TKE. For Reynolds number 1000, spiral COR performed slightly less good than Cartesian COR, but this difference was not seen for Reynolds number 2000. Errors in interpolation between the different grids may have resulted in higher RMSE.

The TE of the spiral measurements was approximately half the TE of the Cartesian measurements. This may be expected to result in around a 50% decrease in spatial misregistration. In the present study, displacement artifacts were, in general, less pronounced in the spiral compared with the Cartesian measurements. However, in the oblique measurements, both the Cartesian and spiral measurements performed poorly in terms of volumetric flow‐rate estimation in the region of the jet. In the oblique measurements, the displacement will cause the signal from spins with high velocities to move toward the edge of the phantom; however, there stills seems to be some remaining signal with high velocities in the center, resulting in a wider jet, and consequently an overestimation of the flow rate. The TE in the spiral measurements might not have been short enough to avoid these effects completely. Postprocessing techniques might be used to reduce the effects of velocity displacement 28.

The scan time of the COR spiral scans was only 23% of the time of the corresponding Cartesian scans. For the TRA scans, the spiral scan time was 39% of the Cartesian scan, which had a slightly smaller field of view. Parallel imaging or longer spiral readouts would further decrease the scan time of spiral 4D flow MRI. Here, the length of the spiral readouts was kept short to obtain a reasonable temporal resolution in an in vivo scan. No off‐resonance correction was deemed necessary, as the spiral readouts were relatively short 12. Alternative spiral readouts, such as spherical stack of spirals, spiral shells, and spiral cones 27 could also further reduce the scan time and have the same benefits of a short TE. While the behavior of a spherical stack of spirals should be similar to the sequence used in this work, more complex 3D readouts may be more sensitive to flow 27 and require longer readouts, which increase the sensitivity to off‐resonance and inhomogeneity. Moreover, a stack of spirals approach allow for a nonisotropic field of view and resolution.

A limitation of this study was that only steady flow was imaged, while in vivo flow is pulsatile. Pulsatile flow may induce more artifacts, as there will be acceleration in both the spatial and temporal domain. Signal‐to‐noise ratio (SNR) of the spiral sequence was not evaluated in this study, but previous studies indicate that a spiral sequence has similar SNR to a Cartesian scan accelerated using SENSE factor 2, but is twice as fast 12. Furthermore, the SNR was boosted using signal averaging to better depict the effects of displacement. While no obvious susceptibility artifacts were observed, this may be a problem for longer spiral readouts or higher field strengths, especially if the vessel, or phantom in this case, is surrounded by air. Moreover, partial volume artifacts are not faithfully reproduced using this phantom, as the plastic wall give no signal. The lack of surrounding tissue outside the phantom may also have degraded the flow rate estimates, as it increases the risk of including noise in the segmentation.

To acquire accurate turbulence estimates, a VENC that is too low to avoid velocity aliasing in a stenotic jet was necessary 23. This problem is less prone in in vivo, as the viscosity of blood is higher, resulting in lower Reynolds numbers, and less intense turbulent velocity fluctuations for the same flow rate. By using a 5‐point dual VENC approach 29, 30, these aliasing artifacts can be avoided. Furthermore, multipoint PC‐MRI and Bayesian analysis have been used to increase the accuracy and dynamic range of turbulence estimates 31.

## CONCLUSIONS

Spiral three‐directional 3D PC‐MRI appears favorable for the assessment of stenotic flow. The spiral sequence was three times faster, and less sensitive to displacement artifacts, when compared with a conventional Cartesian sequence. However, flow‐rate estimates in the jet from both methods seem to be sensitive to displacement in some directions. Turbulent kinetic energy obtained with spiral 3D PC‐MRI agreed well with Cartesian data and CFD. Moreover, maximum velocity and volume flow rate values from the spiral and Cartesian data agreed well.
